# Prescriber adherence to treatment guidelines for monoclonal antibodies against Calcitonin Gene-Related Peptide in migraine prophylaxis – a register-based cohort study

**DOI:** 10.1186/s10194-026-02322-1

**Published:** 2026-03-10

**Authors:** Sofia Nordenmalm, Rickard E. Malmström, Sara Freyland, A. Ingela M Nilsson Remahl, Karin Wirdefeldt, Isabella Ekheden

**Affiliations:** 1 Department of Medicine Solna, Division of Clinical Epidemiology, Karolinska Institutet, Stockholm, SE-171 76 Sweden; 2 Medical Diagnostics Karolinska, Clinical Pharmacology, Karolinska University Hospital, Stockholm, SE-171 64 Sweden; 3 Division of Biostatistics, Institute of Environmental Medicine, Karolinska Institutet, Stockholm, SE-171 77 Sweden; 4 Department of Clinical Neuroscience, Karolinska Institutet, Stockholm, SE-171 77 Sweden; 5 Department of Neurology, Karolinska University Hospital, Stockholm, SE-171 64 Sweden; 6 Department of Medical Epidemiology and Biostatistics, Karolinska Institutet, Stockholm, SE-171 77 Sweden; 7 Department of Laboratory Medicine, Division of Clinical Pharmacology, Karolinska Institutet, Huddinge, SE-141 52 Sweden

**Keywords:** Migraine disorders, Calcitonin gene-related peptide, Calcitonin gene-related peptide receptor antagonists, Antibodies, monoclonal, Managed care programs, Practice guidelines as topic, Practice patterns, physicians’, Drug prescriptions, Neurologists, Sweden

## Abstract

**Background:**

Calcitonin Gene-Related Peptide targeting monoclonal antibodies (CGRP mAbs) were introduced for migraine prophylaxis in Sweden through the National Joint Introduction process to ensure an equal, cost-effective, and appropriate national use. This study aimed to assess prescriber adherence to treatment guidelines and evaluate patient treatment persistence during the national introduction of CGRP mAbs.

**Methods:**

In this register-based cohort study, we followed individuals with a migraine diagnosis or dispensation of migraine drugs within Stockholm County from July 2018 to June 2022. We evaluated the guidelines for CGRP mAb treatment (erenumab, fremanezumab, or galcanezumab) according to four categories: 1) patient eligibility, 2) prescriber qualifications, 3) treatment evaluation, and 4) monitoring/reporting. We used a Sankey diagram to illustrate treatment switching and discontinuation patterns and employed boxplots along with Kaplan-Meier estimates to assess the persistence to CGRP mAbs.

**Results:**

Among 93,263 eligible individuals, 2266 (2%) had at least one CGRP mAb dispensation. Most (90%) had a prior migraine diagnosis at the first CGRP mAb dispensation, and 66% had been dispensed at least two prior prophylactic drugs against migraine. In nearly all cases (97%), CGRP mAb prescriptions were issued by neurology or headache specialists. Switching between CGRP mAbs was common and treatment persistence declined across successive sequences. Overall median treatment duration was 5.8 months (interquartile range: 2.8–11.9 months) and was longer for erenumab than fremanezumab and galcanezumab in sequence 1, but not in sequence 2. Kaplan–Meier analysis revealed a 12-month persistence of 58% in sequence 1, 53% in sequence 2, and 46% in sequence 3. A higher persistence was observed for fremanezumab and galcanezumab compared with erenumab in sequence 1, while estimates were similar in sequence 2.

**Conclusion:**

Prescribers within Stockholm County adhere well to CGRP mAb treatment guidelines, with dispensations largely supported by a sound medical rationale, indicating appropriate integration into clinical practice. Guidance on switching between CGRP mAbs is limited and our findings show frequent switching and reduced persistence across sequences. These insights can help clinicians avoid unnecessary switches and optimize migraine care and resource use.

**Graphical abstract:**

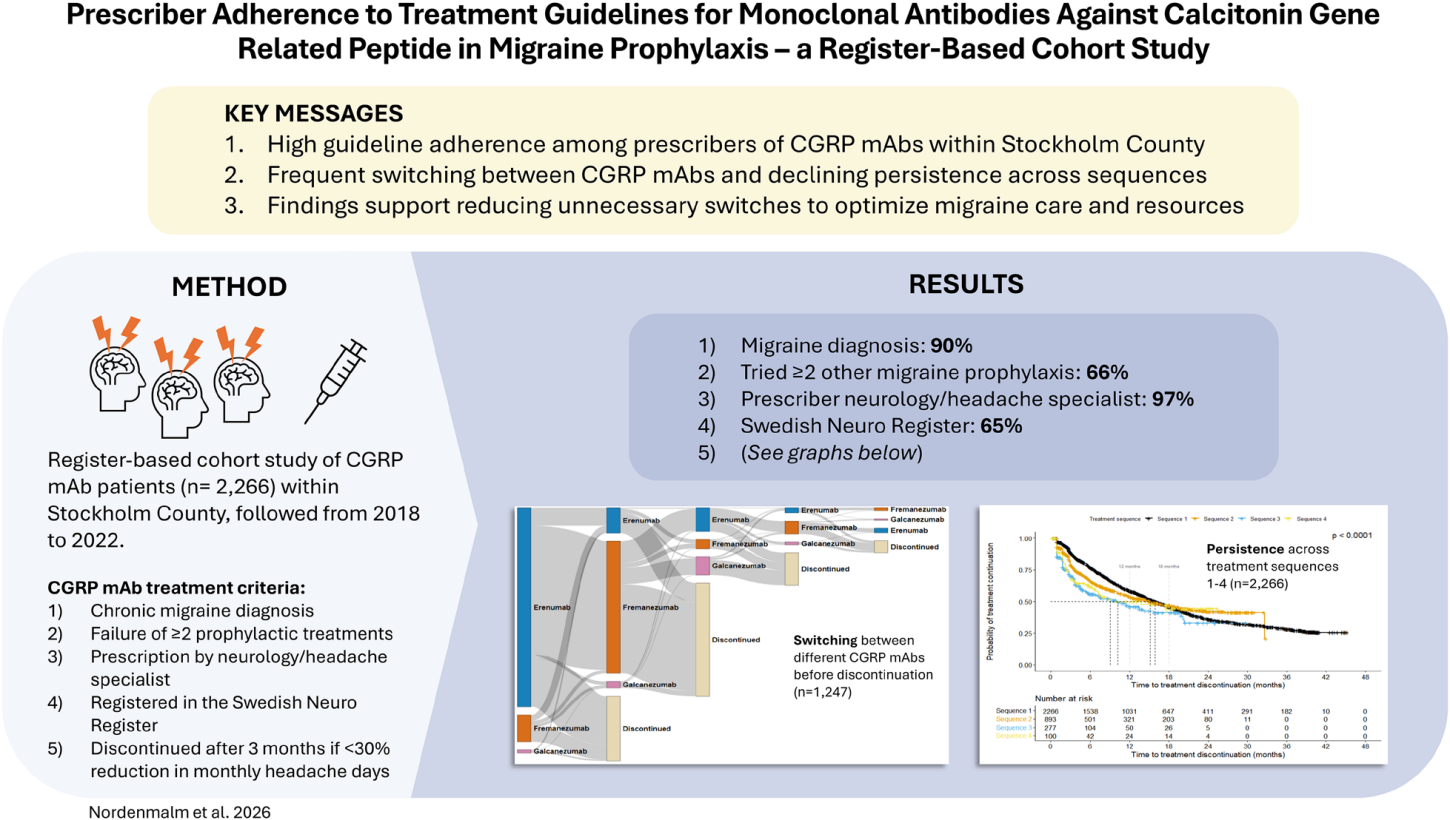

**Supplementary Information:**

The online version contains supplementary material available at 10.1186/s10194-026-02322-1.

## Simple summary/Highlights

We found a high adherence to national treatment guidelines among prescribers within Stockholm County, supporting a sound medical rationale for CGRP mAb initiation and follow-up.

Switching between CGRP mAbs, especially between erenumab and fremanezumab, despite limited national guidance on switching strategies, was common and treatment persistence declined across successive treatment sequences.

Our findings can help guide clinicians in limiting unnecessary treatment switches, ensuring more effective and resource-conscious migraine care.

## Introduction

Migraine is one of the most disabling neurological disorders, ranked the eighth leading cause of health loss in the global population disease burden measured as years lived with disability (YLD) [1, 2]. Disability caused by migraine affects over 1 billion individuals globally [1, 2], including more than 1 million individuals in Sweden [3]. Migraine is classified into episodic and chronic forms, where chronic migraine is considered the most severe subtype [4], with a global prevalence of 1–2% [5]. Acute and prophylactic pharmacological treatment for migraine is widely used, with previous studies reporting prevalence rates of 50–90% among patients [6, 7]. The range of prophylactic drugs for chronic migraine – such as beta blockers, amitriptyline, topiramate, and onabotulinumtoxin A (BTX-A) – was expanded with the approval of monoclonal antibodies that target Calcitonin Gene-Related Peptide (CGRP mAbs) in 2018.

After national reimbursement of the CGRP mAbs erenumab, fremanezumab, and galcanezumab under the National Drug Benefit Scheme in 2019–2020, use expanded quickly, driven by previously unmet clinical needs. By 2022, about 6400 individuals (0.61 per 1000) had been dispensed CGRP mAbs, with large regional variation – Stockholm County had the highest prevalence, 0.90 per 1000, compared to 0.23 in the county with the lowest prevalence [8].

To ensure an equal, cost-effective, and appropriate use of new drugs across all healthcare regions, CGRP mAbs were included in the National Joint Introduction process [9, 10]. The national recommendations, first published in January 2019, advised the following: 1) *Patient Eligibility*: CGRP mAbs should be prescribed exclusively to patients with chronic migraine, defined as experiencing headaches on 15 or more days per month, of which at least 8 are migraine days. Eligible patients should have failed or been unable to tolerate at least two prior prophylactic drugs; 2) *Qualified Prescribers*: Prescriptions should be issued by neurologists or physicians practicing in specialized migraine clinics; 3) *Treatment Evaluation*: Treatment response should be assessed after three months and discontinued if there is no effect or less than 30% reduction in number of headache days. Even for responders, therapy should be time-limited, with a recommended break after 12–18 months. Switching between CGRP mAbs or combining with onabotulinumtoxin A (BTX-A) was not addressed in the guidelines; 4) *Monitoring and Reporting*: Patients should be registered in the Swedish Neuro Register for Severe Neurovascular Headache and are required to self-report their migraine symptoms to the register [11].

Despite the widespread adoption of CGRP mAbs, studies examining prescriber adherence to treatment guidelines, such as those outlined in the National Joint Introduction, remain limited. Previous studies on physician adherence to migraine treatment guidelines, predating the introduction of CGRP mAbs, have reported low compliance with triptan recommendations in Denmark [12], guideline-concordant preventive choices in Spain [13], and increasing adherence over time for both acute and preventive treatments among U.S. veterans [14]. To the best of our knowledge, no previous population-based study has examined prescriber adherence to national CGRP mAb-specific recommendations.

Taking advantage of a population-based database covering the entire Stockholm County population, our primary objective was to assess adherence to these guidelines and evaluate treatment persistence during the National Joint Introduction of CGRP mAbs. Additionally, we compared migraine-related health care utilization and consumption of migraine prophylactic treatments between individuals who received CGRP mAbs and those who did not.

## Materials and methods

### Study population and design

This was a register-based cohort study with data originating from routine health care visits registered in the VAL database from January 1993 to June 2022 within Stockholm County, Sweden. The study base consisted of approximately 2.4 million inhabitants, representing 23% of the total population in Sweden in 2022. Individuals were followed from the index date until the earliest of the following: end of the study period (June 9, 2022), emigration from Stockholm County, or death. The CGRP mAb eptinezumab for intravenous infusion, and small-molecule CGRP receptor antagonists (gepants) were not included because they were authorized either after or very close to the date of data extraction (June 9, 2022).

The VAL database, which has been widely used in previous research [15, 16], covers all consultations in publicly subsidized primary care and specialist outpatient care, along with all hospitalizations for residents of Stockholm County. Individual-level data on specialist outpatient care and hospitalizations have been available since 1993, while data on primary care have been available since 2003 and from the National Prescribed Drug Register from July 2010 and onwards. The data extraction was conducted by the Center for Health Data (Centrum för Hälsodata) at Region Stockholm, the regional public body responsible for healthcare within Stockholm County, on June 9, 2022.

### Data collection

Information on demographic variables, including age, sex, vital status, and regional immigration/emigration data (available from 1995), was collected. Diagnoses based on the International Classification of Diseases (ICD) were also obtained. Information on drugs administered during healthcare visits (e.g., BTX-A) was also included.

Dispensations of CGRP mAbs during the study period were identified using ATC codes (erenumab N02CD01, reclassified from N02CX07 in 2020; galcanezumab N02CD02, reclassified from N02CX08; and fremanezumab N02CD03). Furthermore, dispensations of drugs categorized as prophylactic migraine drugs were identified using the following ATC codes: metoprolol (C07AB02), propranolol (C07AA05), candesartan cilexetil (C09CA06), amitriptyline (N06AA09), and topiramate (N03AX11). Data on exposure to BTX-A (M03AX01) was obtained through ATC code registrations from healthcare visits. No dispensation data were used for BTX-A, as this drug is typically purchased directly by the migraine clinic and administered on site.

Unique workplace codes were used to classify the qualification of the prescriber as *Neurology or Headache*. Specialty Care workplace codes with specialties such as ‘Anesthesia’, ‘Internal Medicine’, ‘General Medicine’, and ‘Rehabilitation Medicine’ were classified as *Possibly Neurology*. Workplace codes were classified as *Other than Neurology* if they had a Specialty of ‘General Medicine’ or ‘Primary Care’, or if they belonged to specialties with no indication that they could be used by neurologists.

Registrations in the quality register *Severe Neurovascular Headache,* a sub-register of the Swedish Neuro Register, was assessed using aggregated data [11]. We extracted the number of individuals recorded as newly initiated on CGRP mAbs (erenumab, fremanezumab, or galcanezumab) within Stockholm County between January 2018 and June 2022. These data were not linked on an individual level to the study population.

### Inclusion criteria

**Fig. 1:**
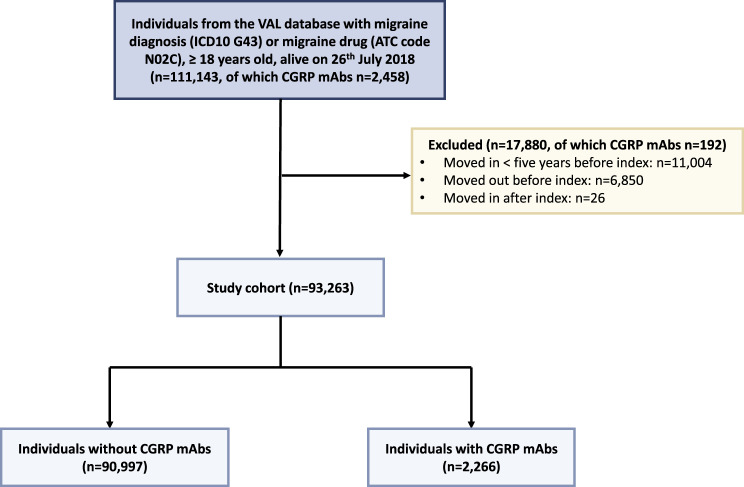
Flowchart illustrating the inclusion and exclusion criteria for the study cohort. *ICD-10* refers to the International Classification of diseases, 10th revision, while *ATC* denotes the Anatomical Therapeutic Chemical classification system

Individuals eligible for inclusion in the study had either: 1) at least one registered diagnosis of migraine (ICD-10: G43, ICD-9: 346) between January 1993 and June 2022, or 2) at least one dispensation of antimigraine drugs (ATC-code N02C) between July 2010 and June 2022 (Fig. 1). Antimigraine drugs available on the Swedish market during the study period included: N02CA Ergot alkaloids; N02CC Selective serotonin (5HT1) agonists (triptans); N02CD CGRP antagonists; and N02CX Other antimigraine preparations (including one herbal drug). Additional inclusion criteria were complete date of birth information, age 18 years or older at the index date, and alive on July 26, 2018 (when the first CGRP mAb was authorized in the EU) (Fig. 1).

### Exclusion criteria

Individuals were excluded from the study population if they had either: 1) moved to Stockholm County less than five years before index date; 2) moved from the county before index date, unless they later moved back more than five years prior to index date; or 3) moved to the county after index date without a prior relocation occurring after index date (this may reflect delays in the registration of national migration data), (Fig. 1).

### Evaluation of treatment guidelines

We categorized the treatment guidelines into four themes: 1) patient eligibility; 2) prescriber qualifications; 3) treatment evaluation; and 4) monitoring and reporting. Each category was evaluated using one or several outcome variables.

### Statistical analyses

Index date was defined based on either CGRP mAb exposure, migraine diagnosis, or dispensation of other N02C-class drugs. For individuals treated with CGRP mAbs, the index date was defined as the date of their first CGRP mAb dispensation. For individuals not treated with CGRP mAbs, the index date was set to the earliest date of either a migraine diagnosis or a dispensation of other N02C-class drugs. If this date occurred before July 26, 2018, the index date was adjusted to July 26, 2018.

Continuous variables were summarized using means with standard deviations or medians with interquartile ranges (IQR), while categorical variables were presented as frequencies and proportions. Differences between categorical variables were tested using chi-square tests, and differences in continuous variables were assessed using independent t-tests or Wilcoxon rank-sum tests, depending on distribution.

### Treatment persistence and discontinuation criteria

We assessed CGRP mAb treatment persistence using the refill-gap method [17] and time to discontinuation. Overlapping refills were ignored, assuming the next dose started on its dispensation date. Days covered per refill were calculated based on SmPC and WHO ATC/DDD; each injection (erenumab, fremanezumab, galcanezumab) was assumed to cover 28 days. The galcanezumab loading dose was not considered.

Discontinuation was defined as either 1) a switch to another CGRP mAb or 2) a gap exceeding the previous supply plus 122 days, occurring between two dispensations, or between the final dispensation and the study end date (June 9, 2022). A treatment sequence was defined as a continuous period without discontinuation. The 122-day grace period reflects dosing intervals and long half-life (~30 days) and aligns with a previous CGRP mAb persistence analysis by the Dental and Pharmaceutical Benefits Agency [18]. Sensitivity analyses used 60- and 180-day grace periods. Please see Supplementary materials for more details on the methodology.

We analyzed the monthly proportion of new users for each type of CGRP mAb in relation to changes in the official list price per syringe over time, as retrieved from the Dental and Pharmaceutical Benefits Agency’s price and decision database.

Data curation and analysis were performed using R (version 4.3.1; R Foundation for Statistical Computing, Vienna, Austria) in RStudio (version 2023.06.1 + 524; RStudio, PBC, Boston, MA, USA) [19]. *p* < 0.05 was considered statistically significant. 

### Use of Generative Artificial Intelligence (AI)

Microsoft 365 Copilot Chat (within the Karolinska Institutet dataprotected environment) was used to improve readability and language, and to obtain suggestions for improving R code. No datasets or personal data were shared with the tool. 

## Results

### Patient characteristics

In the final study population of 93,263 migraine individuals, 2266 individuals (2%) had at least one CGRP mAb dispensation (Fig. 1, Table 1). Individuals with CGRP mAb dispensations were more likely to be women, have a registered diagnosis of migraine, have more migraine-related healthcare visits, and have more dispensations of other migraine prophylactic drugs compared with individuals without CGRP mAb dispensations.

**Table 1:** Characteristics of migraine individuals with and without dispensation of CGRP mAbs (*n* = 93,263)

	No CGRP mAbs (n = 90,997)	CGRP mAbs (n = 2,266)	*P-***value**^**1**^
**Demographic variables**			
**Women, N (%)** **Age at index date,** yrs	67,818 (75%)	1,852 (82%)	<0.001
median (IQR)	47.0 (35.0–58.0)	47.1 (39.0–55.0)	0.2
mean (SD)	47.3 (16.0)	47.1 (11.8)	
**Age group at index date,** yrs, N (%)			<0.001
18–29	13,437 (15%)	174 (8%)	
30–39	16,944 (19%)	393 (17%)	
40–49	21,256 (23%)	736 (32%)	
50–59	18,978 (21%)	632 (28%)	
60+	20,382 (22%)	331 (15%)	
**Health care consumption**			
**Migraine diagnosis,** N (%)			
ever	61,105 (67%)	2,103 (93%)	<0.001
**Number of migraine-related health care visits for those with a migraine diagnosis**			
median (IQR)	2.0 (1.0–4.0)	22.0 (10.0–39.0)	<0.001
mean (SD)	4.1 (7.7)	28.4 (26.5)	
missing	29,892	163	
**Number of migraine-related health care visits for those with a migraine diagnosis,** N (%)			<0.001
<5	48,903 (80%)	272 (13%)	
5–10	7,434 (12%)	265 (13%)	
>10	4,768 (7.8%)	1,566 (74%)	
missing	29,892	163	
**Drug consumption**			
**Number of different types of CGRP mAbs tested,** N (%)			
0	90,997 (100%)	0 (0%)	
1	0 (0)	1,468 (65%)	
2	0 (0)	667 (30%)	
3	0 (0)	121 (5.3%)	
**Other migraine prophylactics**^**2**^, N (%)			
median (IQR)	0.0 (0.0–1.0)	3.0 (2.0–3.0)	<0.001
mean (SD)	0.6 (0.9)	2.6 (1.3)	
0	53,003 (58%)	135 (6.0%)	
1	24,320 (27%)	335 (15%)	
2	9,863 (11%)	635 (28%)	
3	3,020 (3.3%)	624 (28%)	
4	659 (0.7%)	368 (16%)	
5	122 (0.1%)	142 (6.3%)	
6	10 (<0.1%)	27 (1.2%)	
**Other migraine prophylactics ≥ 2,** N (%)	13,674 (15%)	1,796 (79%)	<0.001

1. Pearson’s Chi-squared test; Wilcoxon rank sum test

2. Including dispensations of metoprolol (C07AB02), propranolol (C07AA05), candesartan cilexetil (C09CA06), amitriptyline (N06AA09), and topiramate (N03AX11), as well as registrations of botulinum toxin type A (BTX-A; M03AX01) from healthcare visits

Note: All variables, except age and sex, are presented as means and medians calculated from the start of the study (January 1993) until the end of follow up

Abbreviations: IQR = interquartile range; SD = standard deviation

### Patient eligibility

#### Diagnosis of migraine

Most individuals with a CGRP mAb dispensation (93%) had at least one documented diagnosis of migraine at some point during the study period. For 2047 of the 2266 (90%) individuals with CGRP mAbs, the first diagnosis of migraine was recorded before their first CGRP mAb dispensation, with 1747 (77%) individuals receiving a diagnosis within the 12 months preceding their first CGRP mAb dispensation.

#### Other migraine prophylactic drugs including exposure to BTX-A

Most individuals with a CGRP mAb dispensation (79%) had been dispensed at least two other migraine prophylactic treatments during the study period, with a median of 3 (IQR 2–3) different types of prophylactics—a statistically significantly larger number compared to individuals without CGRP mAbs (Table 1). In total 1503 (66%) of the CGRP mAb-treated individuals had been dispensed at least two other migraine prophylactic drugs before their first CGRP mAb. Only 135 (6%) of the CGRP mAb-treated individuals had not been dispensed any other prophylactic treatment during the study period.

**Fig. 2:**
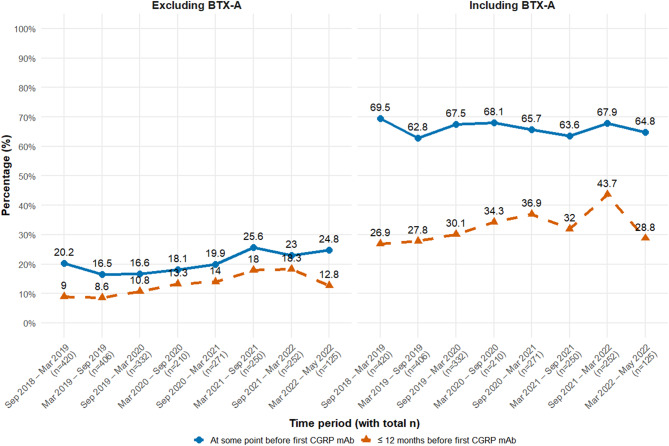
Proportion of individuals dispensed at least two other types of migraine prophylactic drugs per 6-month periods, stratified by BTX-A exposure. Blue solid line represents individuals who received the drugs at any time before the first CGRP mAb, while red dashed line represents individuals who received the drugs within 12 months before the first CGRP mAb

Inclusion of BTX-A substantially increased the proportion of individuals with ≥2 prophylactics prior to CGRP mAb initiation (Fig. 2). For those who received ≥ 2 prophylactics within 12 months before CGRP mAb, the proportion showed a consistent upward trend across most periods in both panels. However, the final period demonstrated a decline, possibly reflecting the shorter observation window (approximately 3 months instead of 6) and smaller sample size (*n* = 125).

Among individuals treated with CGRP mAbs, 1283 (57%) had been exposed to BTX-A at some point during the study period. Of these, 1115 (49%) were exposed before the first CGRP mAb dispensation and 479 (21%) received the treatment between their first and last CGRP mAb dispensation. In contrast, among those without a CGRP mAb dispensation (*n* = 90,997), only 2813 (3%) had been treated with BTX-A. A sensitivity analysis of the annual proportion of individuals with CGRP mAb treatment who also received BTX-A between 2017 and 2022 did not show a statistically significant change during this study period (Supplementary Figure. 1).

#### Qualified prescribers

Among individuals with a CGRP mAb dispensation, 97% obtained both their first and last dispensed prescriptions from prescribers whose workplace codes were classified as ‘Neurology or Headache’. The remaining individuals received their first and last prescriptions from primary care or prescribers with non-neurology specialty care workplace codes, classified as ‘Possible Neurology’ or ‘Other than Neurology’. Only about 2% of the CGRP mAb-treated individuals obtained their first or last dispensed prescription from primary care. Of all CGRP mAb dispensations (*n* = 36,283), 0.3% were issued outside the Swedish National Drug Benefit Scheme. Most of these nonbenefit dispensations occurred in 2018, declining to 0% by the end of the study.

### Treatment evaluation – persistence

#### CGRP mAb type distribution across sequences

Among the 2266 CGRP mAb-treated individuals, 71% initiated treatment with erenumab (the first CGRP mAb approved), 27% with fremanezumab, and 2.6% with galcanezumab. In the second sequence (*n* = 893), fremanezumab dominated (81%), while erenumab and galcanezumab accounted for 15 and 4%, respectively. In the third sequence (*n* = 277), galcanezumab usage increased to 36%, erenumab to 46%, and fremanezumab decreased to 18%. In the fourth sequence (*n* = 100), fremanezumab was again the most frequently used (70%), with erenumab and galcanezumab each representing 15%.

#### Overall persistence

**Fig. 3:**
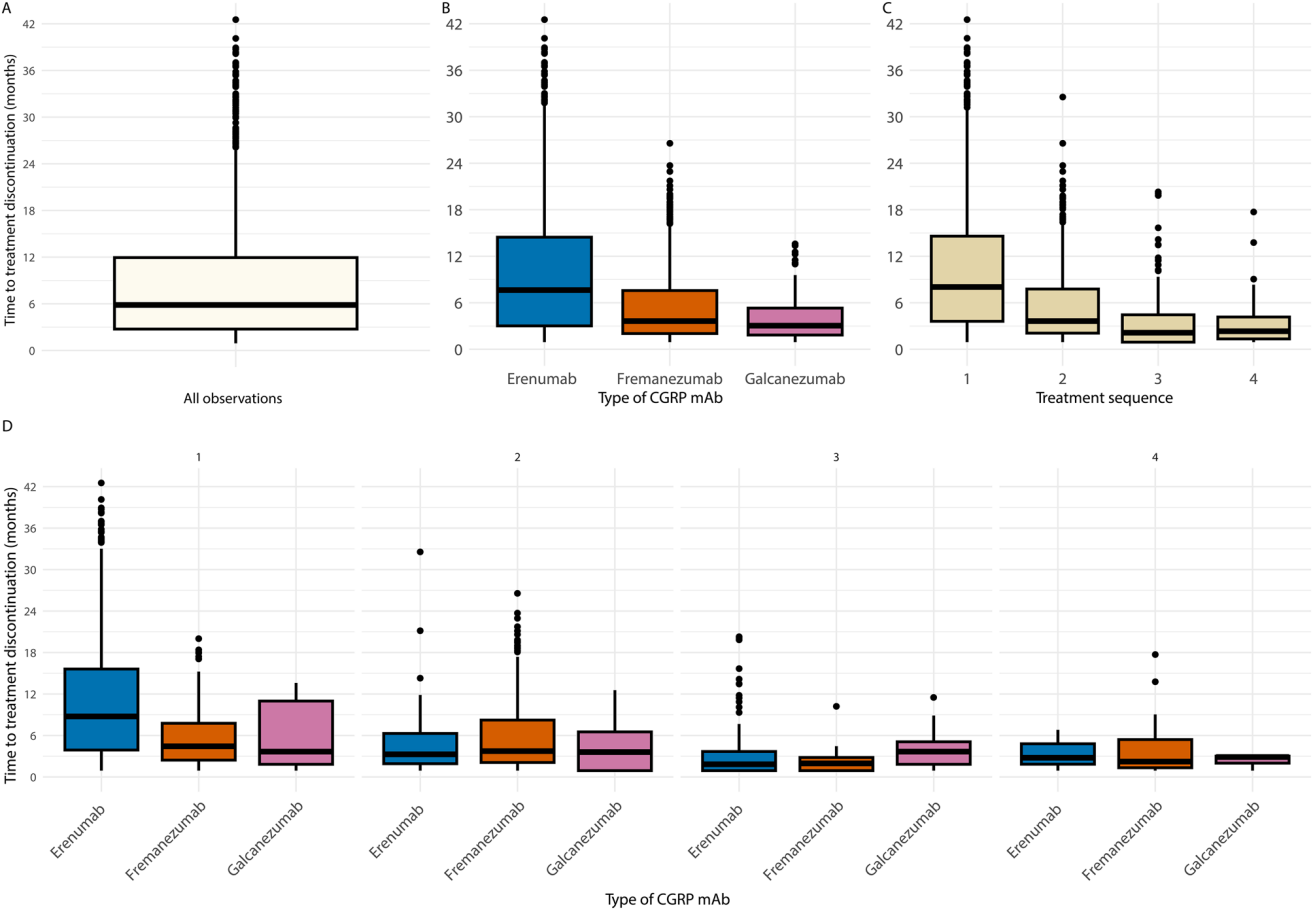
Boxplots illustrating the distribution of time to treatment discontinuation of CGRP mAbs for those individuals who had experienced at least one treatment discontinuation (*n* = 1,247): **A**) for all CGRP mAbs and treatment sequences; **B**) for each type of CGRP mAb in all treatment sequences; **C**) for all CGRP mAbs in the first four treatment sequences; and **D**) for each type of CGRP mAb and for each treatment sequence (the first four). The thick horizontal line inside each box represents the median time to treatment discontinuation. The boxes extend from the first quartile (Q1) to the third quartile (Q3). The interquartile range (IQR = Q3–Q1), which covers the middle 50% of the data. The lines (whiskers) extending from the boxes indicate the range of data within 1.5 times the IQR from Q1 and Q3. The small points outside the whiskers represent outliers, which are values that fall beyond 1.5 times the IQR from the quartiles

Among all CGRP mAb-treated individuals (*n* = 2,266), 1247 (55%) experienced at least one treatment discontinuation, while 1019 (45%) did not. Among individuals with a discontinuation in the first sequence, 52% switched to another CGRP mAb type, 19% discontinued due to a prolonged gap between dispensations, and 28% completely discontinued CGRP mAb treatment. Across all treatment sequences combined, the overall persistence to CGRP mAb dispensations measured by the mean (SD), was 8.4 months (7.6), and by the median (IQR) 5.8 months (2.8–11.9) (Fig. 3A). Of those who discontinued (*n* = 1,247), 77% remained persistent to CGRP mAb for up to 12 months, 36% were persistent for more than 12 months, 18% for more than 18 months, and 7% for more than 24 months.

#### Persistence by CGRP mAb type

When examining CGRP mAb type for all treatment sequences combined (*n* = 1,247), persistence was most pronounced for erenumab (mean 9.9 months (SD 8.2); median 7.6 months (IQR 3.0–14.5)), followed by fremanezumab (mean 5.6 months (SD 4.9); median 3.6 months (IQR 2.0–7.6)) and galcanezumab (mean 4.2 months (SD 3.3); median 3.0 months (IQR 1.8–5.3)) (Fig. 3B). A Kruskal–Wallis test indicated a significant difference in time to treatment discontinuation among the three CGRP mAb groups (*p* < 0.001). Post-hoc Dunn’s tests with Bonferroni correction revealed that erenumab differed significantly from fremanezumab (*p* < 0.001) and galcanezumab (*p* < 0.001), whereas fremanezumab and galcanezumab did not differ significantly (*p* = 0.27).

#### Persistence by treatment sequence

Among those with discontinuation (*n* = 1,247), considering all CGRP mAb types combined, persistence steadily declined with each subsequent treatment sequence (Fig. 3C). In the first sequence, mean persistence was 10.2 months (SD 8.1), and median was 8.0 months (IQR 3.6–14.6), whereas in the second sequence, mean decreased to 5.8 months (SD 5.2), with a median of 3.6 months (IQR 2.1–7.8). There was a significant difference among the four sequences (*p* < 0.001). Dunn’s post-hoc test indicated that all pairwise comparisons were statistically significant (*p* < 0.05) except between sequence 3 and 4 (*p* = 1.00). The mean number of treatment sequences was 2.3 (SD 2.1).

#### Persistence by CGRP mAb type and treatment sequence

When comparing CGRP mAb types across multiple treatment sequences (Fig. 3D), persistence with erenumab was longer than with fremanezumab in the initial sequence (mean 10.8 months (SD 8.3) and median 8.8 months (IQR 3.9–15.6)) vs mean 5.8 months (SD 4.5) and median 4.4 months (IQR 2.4–7.8)), but shorter in the second (mean 5.1 months (SD 5.3) and median 3.3 months (IQR 1.9–6.3) with erenumab vs mean 5.9 months (SD 5.3) and median 3.7 months (IQR 2.1–8.2) with fremanezumab).

For sequence 1, persistence differed among CGRP mAb groups (*p* < 0.001). Dunn’s post-hoc test showed that persistence with erenumab differed statistically significantly from fremanezumab (*p* < 0.001) and galcanezumab (*p* = 0.02), whereas persistence with fremanezumab and galcanezumab did not differ (*p* = 1.00). For sequence 3, persistence also differed among CGRP mAb groups (*p* = 0.014). Dunn’s test indicated that erenumab differed statistically significantly from galcanezumab (*p* = 0.013), while no other pairwise comparisons were statistically significant. There were no statistically significant differences in sequence 2 and 4.

Sensitivity analyses using grace periods of 60 and 180 days, compared to the primary analysis (122 days), showed generally consistent and statistically significant differences in persistency across all comparisons, except for sequence 3, where no differences were observed among the CGRP mAb types (Supplementary Figures 2 and 3).

#### Sankey plot

**Fig. 4:**
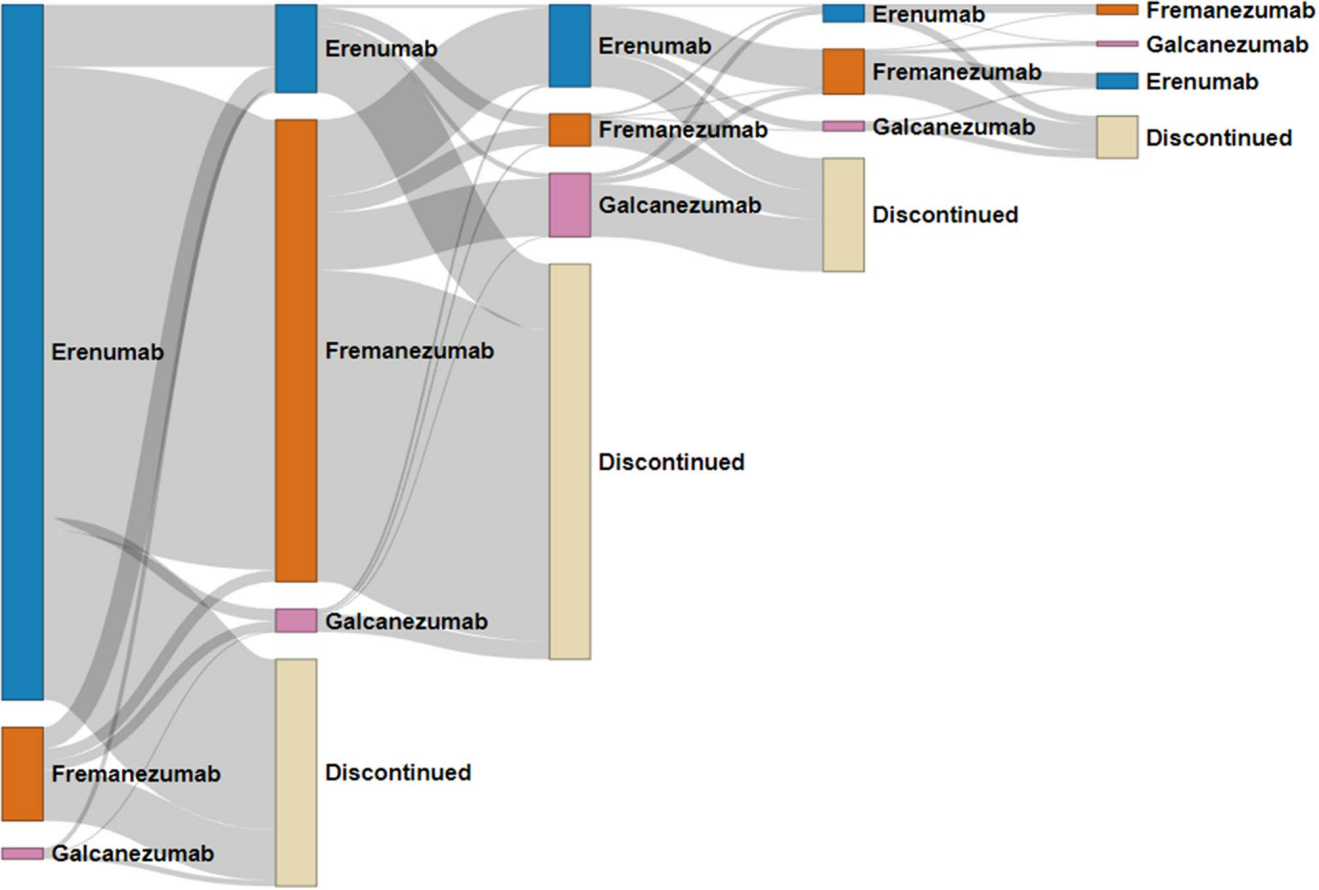
Sankey diagram illustrating the flow of individuals across CGRP mAb types during the five first treatment sequences. The analysis includes only individuals who experienced at least one treatment discontinuation (*n* = 1,247). Nodes in the first column represent sequence 1, those in the second column represent sequence 2, and so on. The width of the connecting lines (links) corresponds to the number of individuals, with thicker lines indicating larger numbers

The Sankey plot (Fig. 4), which includes individuals with at least one treatment discontinuation during the study period (*n* = 1,247), showed that switching between different types of CGRP mAbs before complete treatment discontinuation was common, particularly between erenumab and fremanezumab.

Among the 1084 individuals who started on erenumab in the first treatment sequence, 702 (65%) switched to fremanezumab in the second sequence, 266 (25%) discontinued CGRP mAb treatment entirely (with no subsequent sequences), while a smaller number of 97 (9%) re-started erenumab. Among the 146 individuals who initiated treatment with fremanezumab, 79 (54%) discontinued CGRP mAb treatment entirely, 33 (23%) switched to erenumab, and a smaller proportion either re-started fremanezumab (*n* = 18, 12%) or switched to galcanezumab (*n* = 16, 11%) in the second sequence. Lastly, of the 17 individuals who began with galcanezumab, 9 (53%) discontinued CGRP mAb treatment entirely.

#### Survival analysis

Of the 2266 CGRP mAb-treated individuals included in the Kaplan-Meier survival analysis, 1247 (55%) discontinued treatment, while 1019 were censored due to ongoing treatment at the end of follow up. No discontinuation events were observed before day 28 (0.92 months), as each CGRP mAb injection covered a 28-day period. Individuals were considered at risk for treatment discontinuation only after this initial coverage period had passed, which explains the clustering of events shortly after day 28.

**Fig. 5:**
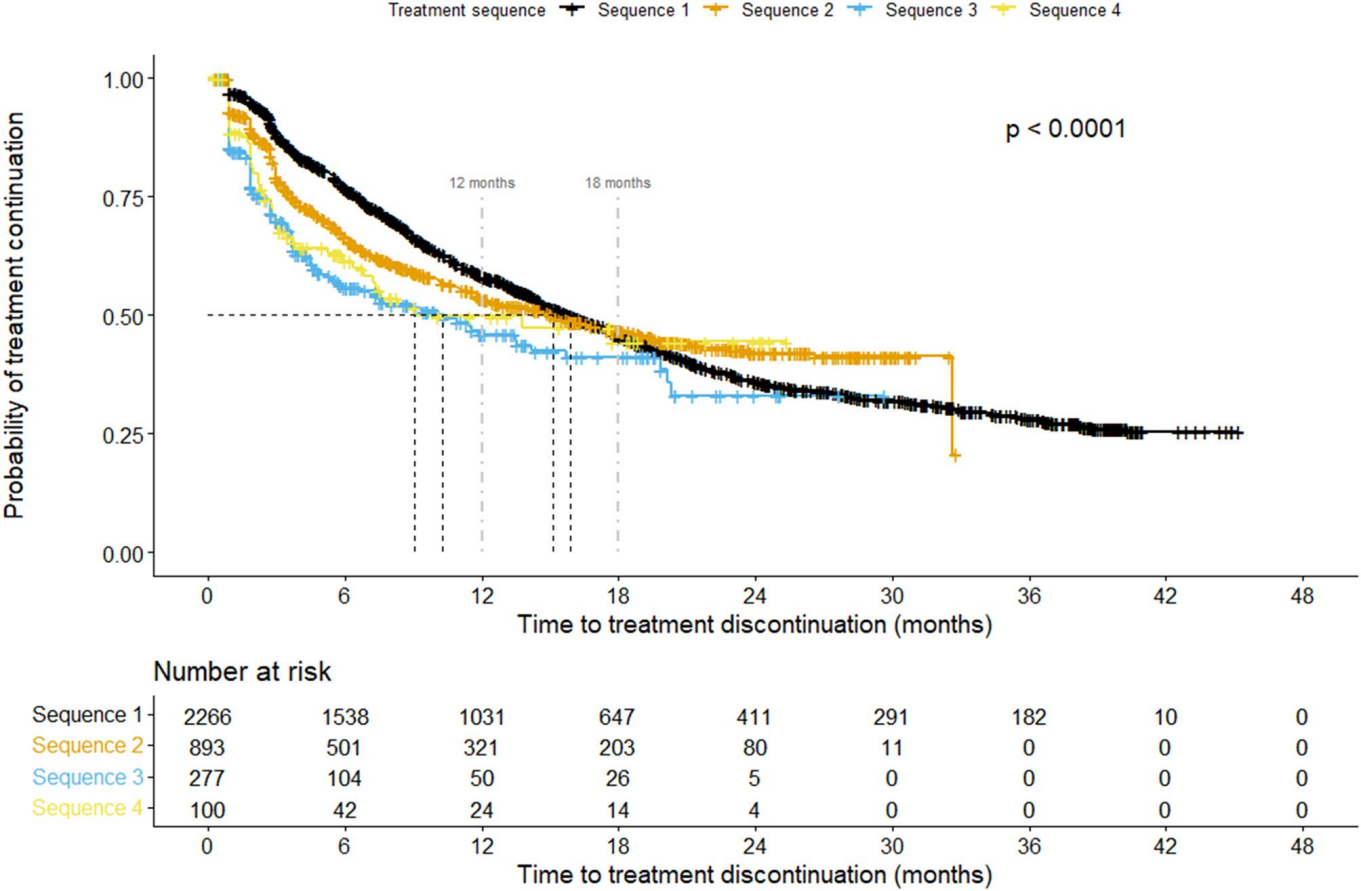
Kaplan Meier survival analyses showing the probability of treatment continuation across up to four treatment sequences among all individuals who initiated a given sequence (*n* = 2,266). Log-rank *p*-value < 0.0001. Individuals without any treatment discontinuation before end of follow up were censured. The black line represents sequence 1, the orange line sequence 2, the blue line sequence 3, and the yellow line sequence 4. Black dashed lines represent the median survival time (50%) for each sequence. Light gray dashed lines at 12 and 18 months indicate the recommended time point for a treatment break following a positive response to CGRP mAbs

Survival analysis of treatment continuation probability across the first four treatment sequences, combining all three CGRP mAb types (Fig. 5), demonstrated a statistically significant, progressive decline with each successive sequence (log-rank test *p* < 0.001), indicating reduced persistence over time—at least during the first 18 months of treatment. The probability of remaining on treatment at 12 months was approximately 58% in sequence 1, 53% in sequence 2, 46% in sequence 3, and 50% in sequence 4. Median survival time was approximately 16 months in sequence 1, 15 months in sequence 2, 10 months in sequence 3, and 9 months in sequence 4.

Notably, the number of individuals at risk declined markedly across sequences: 2266 in sequence 1, 893 in sequence 2, 277 in sequence 3, and 100 in sequence 4. The proportion of censored observations increased from 45% in sequence 1 to 53% in sequences 2 and 3, and 56% in sequence 4. In Sequence 2, the Kaplan–Meier curve shows a sharp decline around month 33. This drop is explained by the very small number of patients remaining in the risk set at that time. When only a few individuals are at risk, even a single event produces a large change in the survival estimate.

Sensitivity analyses using alternative grace periods (60 and 180 days) showed the same overall pattern of progressively reduced persistence across treatment sequences (Supplementary Figures 4 and 5). As expected, the grace period influenced the estimated duration of persistence: a shorter grace period (60 days) resulted in slightly shorter median survival times, while a longer grace period (180 days) produced slightly longer estimates. This reflects the definition of discontinuation where stricter criteria classify more patients as having discontinued earlier, whereas more lenient criteria allow longer gaps before treatment is considered ended. Importantly, these variations did not alter the statistical significance or the relative ranking of treatment sequences.

A positive response to CGRP mAbs should be time-limited, with a treatment break recommended after 12–18 months. Therefore, persistence in the survival analysis, when interpreted in relation to guideline adherence, can only be meaningfully assessed up to this timeframe (indicated in Fig. 5). Beyond 18 months, discontinuation may reflect either physician compliance with treatment recommendations or patient preference, making interpretation uncertain.

**Fig. 6:**
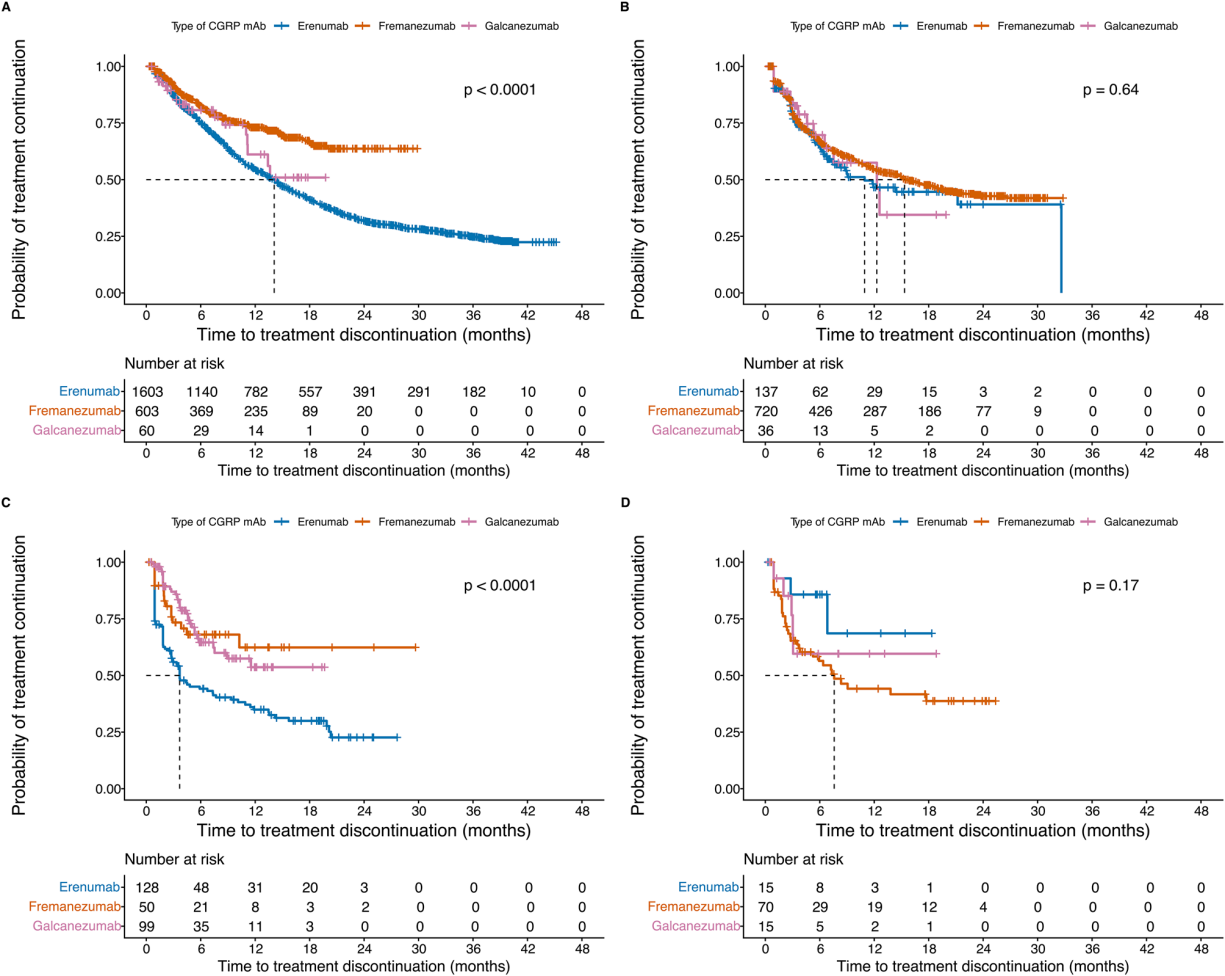
Kaplan Meier survival analyses showing the probability of treatment continuation per type of CGRP mAb for **A**) treatment sequence 1 (*n* = 2,266), log-rank *p*-value < 0.0001; **B**) treatment sequence 2 (*n* = 893), log-rank *p*-value = 0.64; **C**) treatment sequence 3 (*n* = 277), log-rank *p*-value < 0.0001; and **D**) treatment sequence 4 (*n* = 100), log-rank *p*-value = 0.17. Individuals without any treatment discontinuation before end of follow up were censored. Blue lines represent erenumab, red lines represent fremanezumab, and pink lines represent galcanezumab. Black dashed lines represent the median survival time (50%) for CGRP mAb

Further, survival analysis of treatment continuation probability by CGRP mAb type across treatment sequences 1–4 (Fig. 6) showed that fremanezumab and galcanezumab had a significantly higher probability of continuation compared with erenumab in sequence 1, while probabilities were similar across treatments in sequence 2. For fremanezumab, the probability of remaining on treatment after six months was 82% in sequence 1, 67% in sequence 2, 68% in sequence 3, and 56% in sequence 4. For galcanezumab, the corresponding probabilities were 81, 70, 65, and 60%, while for erenumab they were 75, 63, 44, and 86%. Sensitivity analyses using grace periods of 60 and 180 days confirmed the overall pattern for sequences 1 and 2 (Supplementary Figures. 6 and 7). However, with the 60-day grace period, patterns for sequences 3 and 4 diverged, likely due to the small number of individuals in these later sequences.

The analysis on the monthly proportion of new users for each type of CGRP mAb in relation to changes in the official list price per syringe over time showed a temporary increase in the proportion of new users initiating galcanezumab and a corresponding decrease in initiations of erenumab around January 2021 (Supplementary figure. 8 and 9).

### Monitoring and reporting

The total number of individuals registered in the Swedish Neuro Register for Severe Neurovascular Headache as newly initiated on CGRP mAbs (erenumab, fremanezumab, or galcanezumab) within Stockholm County between January 2018 and June 2022 was 1470 (aggregated data) [3]. Dividing this number by the 2266 individuals with CGRP mAb dispensations in our study population resulted in an estimated coverage of the register of approximately 65%. Notably, we did not link the individuals in the register to those in our study population.

## Discussion

### Main findings on adherence to treatment guidelines

In this study, we evaluated adherence to national treatment guidelines for CGRP mAbs in a Swedish region with high dispensation rates. Altogether, our findings indicate that prescribers within Stockholm County adhere well to the guidelines. While improvement is possible, the high number of dispensations appears medically justified.

To the best of our knowledge, this is the first real-world study to assess prescriber adherence to national CGRP mAb-specific recommendations. Our findings provide important insights into how these guidelines are applied in practice. Importantly, our results are consistent with the goals of Sweden’s National Joint Introduction, a framework designed to ensure equal, cost-effective, and appropriate use of new therapies nationwide. High dispensation rates, combined with generally good adherence to guidelines, suggest that clinical decisions largely follow this framework, although some deviations remain.

Most individuals receiving CGRP mAbs had a registered migraine diagnosis. Because ICD codes for chronic migraine are lacking, BTX-A treatment serves as a practical proxy. In our study, 57% of individuals with CGRP mAbs had BTX-A exposure at some point, compared with 3% among those without CGRP mAbs. This indicates that chronic migraine is substantially more prevalent among CGRP mAb-treated individuals compared to the broader migraine population.

Prior exposure to at least two other migraine prophylactics was frequent (66% at some point before CGRP mAbs; 30% within 12 months), yet lower than expected given recommendations, indicating prescribing deviations from the recommendations. Yet, the proportion was higher in this study than in Austria (11.4%) [20] noting that Austrian data on prior prophylactics were limited to one year preinclusion. While Sweden’s healthcare system ensures universal access and promotes social equity, migraine care remains unequitable [21]. Constrained resources in primary care [22], often representing the first point of contact for migraine patients, combined with a lack of neurologists, who are authorized to prescribe advanced migraine prophylactic treatments, may delay referral. Consequently, neurology/headache specialists may initiate CGRP mAbs even without trials of two prior prophylactics.

Although it was reassuring that most individuals obtained their CGRP mAb prescription from a neurology/headache specialist and were monitored in the national quality register, the disparities in migraine are also evident across socioeconomic groups. Our recent study [23] found that higher socioeconomic status was associated with CGRP mAb dispensation within Stockholm County, highlighting unequal access. To address these gaps, the Swedish government has tasked the National Board of Health and Welfare with developing national guidelines aimed at improving accessibility and equity in migraine care [21].

Sweden’s system of highcost protection for both outpatient care and prescription drugs [22] likely mitigates, but does not eliminate, these disparities. Outpatient fees are capped at a relatively low 12-month maximum (1,100–1,200 SEK during 2018–2022), and prescription drugs covered by the National Drug Benefit Scheme (including CGRP mAbs since 2019–2020) become free of charge once individuals reach the 12-month pharmaceutical cost threshold (2,250–2,400 SEK during 2018–2022). These financial protections substantially reduce outofpocket costs, making it unlikely that patient fees are the primary driver of treatment differences. Utilization is more plausibly shaped by systemlevel factors such as national guideline recommendations, drug availability, and variation in specialist access. Nevertheless, financial influences cannot be entirely excluded, as a single dispensation of a CGRP mAb represents a substantial onetime cost that would immediately bring patients to the pharmaceutical cost threshold.

Taken together, our findings underscore the need for continued monitoring of how new therapies are integrated into practice. Regional differences in uptake and adherence may reflect local interpretations of recommendations, patient needs, or resource constraints.

### Treatment persistence and switching

We found that treatment persistence declined across successive CGRP mAb sequences, and that switching, particularly between erenumab and fremanezumab, was common prior to cessation. This pattern aligns with the Swedish Dental and Pharmaceutical Benefits Agency’s report [18] of longer-than-expected treatment durations as well as frequent switching rather than discontinuation. Our 12-month persistence estimate (58% for the first CGRP mAb) is broadly consistent with previous evidence [20, 24], supporting the robustness of our findings.

Our results further suggest that while some individuals remain on their first CGRP mAb long-term, discontinuation occurs earlier in subsequent sequences, consistent with a prior study [25] reporting higher treatment persistence at 12 months for the first CGRP mAb (67%) compared to the second (50%) and third (37%). The same study also demonstrated that the initial anti-CGRP treatment achieved the highest response rate (≥50% reduction in monthly migraine days), although some patients benefited from switching strategies. Similar trends were observed in Ireland using a managed access protocol [26] where persistence beyond 12 months exceeded 75% in patients failing ≥ 3 prior preventives.

Compared with an Austrian nationwide study [20] (2018–2022, *n* ≈ 9,000) reporting 42.9% persistence ≥ 1 year and median survival of 11 months for the first sequence and 7 months for later ones (Kaplan–Meier, 60-day grace period), our analysis (122-day grace period) showed somewhat longer persistence: 16, 15, and 10 months for sequences 1–3 (sensitivity: 13, 10, and 7 months). In Austria, treatment breaks were common, with 54% restarting the same CGRP mAb and 17% switching.

Persistence varied across CGRP mAb types and treatment sequences. Erenumab had the longest completed median duration in sequence 1 but was surpassed by fremanezumab in sequence 2, as illustrated in boxplots. In contrast, Kaplan–Meier analysis, which accounts for censored data, revealed a higher probability of treatment continuation for fremanezumab and galcanezumab than erenumab in sequence 1, possibly reflecting patient selection, dosing convenience (quarterly fremanezumab), and tolerability (constipation more common with erenumab). Probabilities were similar across CGRP mAbs in sequence 2, suggesting diminishing influence of drug type. The discrepancy between boxplots and survival curves illustrates how completed durations differ from persistence probabilities over time, especially when censoring is considered. Interpretation of galcanezumab results is limited by small sample size (*n* = 60, 2.6%). A previous study [27] with more galcanezumab initiators (35%) reported lower discontinuation rates for galcanezumab compared to fremanezumab and erenumab over 12 months (48% vs. 52%, *p* = 0.005; and 50%, *p* = 0.040).

The timing of market availability, price differences, and reimbursement likely amplified these patterns. Erenumab, approved July 2018 and reimbursed January 2019, was the sole reimbursed option for nearly a year and the index drug for 71% of individuals. Fremanezumab, (approved March 2019) was reimbursed in November 2019 and galcanezumab (approved November 2018) in August 2020. This staggered timeline meant that early adopters had limited alternatives, which may explain erenumab’s longer median duration in sequence 1, as illustrated in boxplots. Conversely, as additional mAbs became available and reimbursed, clinicians could switch more readily in cases of suboptimal response or tolerability, contributing to shorter median durations for drugs made available later. Over time, improved clinical experience may also have contributed to better persistence among later entrants.

When the first national guideline was issued in January 2019, erenumab (Aimovig) was the only CGRP mAb included in the Swedish National Drug Benefit Scheme and was therefore recommended for eligible patients. Fremanezumab (Ajovy) was added in November 2019 as an equivalent option to erenumab. Both products were covered by confidential national rebate agreements, creating a difference between the public list price and the actual net price. Galkanezumab (Emgality) was incorporated into the guidelines in September 2020 and recommended as the first-line option because its lower public list price (without a confidential rebate) resulted in a cost per Quality-Adjusted Life Year (QALY) comparable to the rebated net prices of erenumab and fremanezumab. In January 2021, following the expiration of rebate agreements and official list price reductions, the recommendation stated that all three mAbs had similar costs, and none was preferred over the others.

Although the period during which galcanezumab was recommended as the firstline option was relatively short (September 2020 to January 2021), the pricing shift was reflected in prescribing patterns. Specifically, we observed a temporary increase in the proportion of new users initiating galcanezumab and a corresponding decrease in initiations of erenumab around January 2021. While this effect was modest and unlikely to have shaped longterm utilization, it illustrates that cost considerations can have immediate, measurable consequences for realworld treatment choices.

Collectively, our findings suggest that treatment persistence with CGRP mAbs is influenced by both drug type, treatment sequence, and timing availability.

### Discontinuation and switching strategies

Reasons for discontinuation remain unclear but may include lack of efficacy, side effects, or adherence to recommended treatment duration [28]. More specifically, case-reports and case-series have raised concerns regarding the long-term safety and negative effects on the cardiovascular system, bone density, hair growth and immunity in the context of preventive CGRP treatment, although safety evaluations in previous clinical trials until present have been reassuring [29]. Frequent switching and discontinuation may reflect ongoing clinical evaluation but could also indicate that individuals with more severe migraine are less likely to benefit from reinitiation or multiple switches. While some observational studies suggest clinical benefit from switching between ligand- and receptor-targeted CGRP mAbs [30–33] with fewer overall and gastrointestinal adverse events when switching from erenumab to fremanezumab [34], robust evidence supporting multiple switches after treatment failure is limited. Our findings raise important questions about the effectiveness of such strategies in non-responders, especially given the high cost and limited healthcare resources.

We believe that the relatively high rate of CGRP mAb continuation and switching may reflect the limited treatment options available once these therapies have been tried. When patients fail to reach the expected 30% efficacy threshold despite exhausting available treatments, including BTX-A, clinicians face the dilemma of what next. In such cases, continuing CGRP mAbs may be perceived as the least inadequate option, even with suboptimal response. In our study, 21% of the 2266 individuals with CGRP mAb dispensations also received BTX-A during the same treatment period, suggesting suboptimal response to CGRP mAb monotherapy.

National guidance on switching between CGRP mAb types has been limited. The Swedish National Joint Introduction recommendations have provided no specific advice, and Region Stockholm’s 2024 guidelines highlight the lack of evidence for trying more than one CGRP mAb [35]. In contrast, recent international guidelines recommend switching after 3–6 months of inadequate response [36].

### Strengths

Swedish residents have access to universal health care coverage with high-cost protection including primary and specialist care and medication costs. Therefore, economic barriers against seeking medical care are relatively low. This provides a substantial advantage when using the VAL database as it captures routinely collected, anonymized data from all publicly subsidized healthcare providers within Stockholm County. Unlike the Swedish National Patient Register [37], VAL includes data from both inpatient, outpatient, and primary care, where most migraine cases are diagnosed and treated. The inclusion of primary care data in this study is particularly important, as general practitioners are the first line of care for migraine patients, and no national register in Sweden currently provides comprehensive coverage of primary care. This broader coverage of the VAL database enabled a more comprehensive analysis of migraine treatment patterns that would otherwise have been limited to inpatient care and specialized outpatient services.

The near complete coverage of specialist care and dispensed drugs is another important strength of this study, because migraine patients are typically referred to specialist physicians for initiation of CGRP mAbs and other preventive therapies after having failed at least two oral prophylactic drugs. Although all physicians can prescribe CGRP mAbs under independent prescribing, only prescriptions by specialist physicians in neurology or headache specialists are reimbursed by The Dental and Pharmaceutical Benefits Agency. All dispensed CGRP mAbs were nevertheless captured in the VAL database and used in this study, regardless of prescriber and reimbursement.

We classified migraine based on either a registered diagnosis of migraine or dispensations of drugs classified under the ATC code N02C (Antimigraine drugs). Given that fewer than 50% of individuals with migraine in Sweden receive a formal diagnosis from a healthcare provider [38], this dual approach was implemented to improve detection of individuals with migraine.

## Limitations

Our study has several limitations. Register data from VAL lack information on treatment indication. Although CGRP mAbs are primarily used for migraine, the absence of a unique ICD code for chronic migraine introduces a risk of misclassification. While we had complete coverage of dispensed CGRP mAbs, we cannot confirm whether the drugs were actually administered or the exact dosing regimen, which may lead to an overestimation of persistence. Clinical outcome data and reasons for discontinuation were also unavailable, limiting interpretation of persistence since discontinuation could reflect insufficient effect, adverse events, or the recommended maximum treatment duration after a positive response.

Furthermore, although we had data on prior prophylactic dispensations up to eight years before index date (July 2010) and at least five years for individuals who moved into the county, earlier use may have occurred, potentially underestimating previous exposure. While most prescriptions originated from neurology or headache clinics (97%), prescriber specialist qualifications could not be verified. Finally, the study was restricted to Stockholm County, which may limit generalizability to other regions or countries with different healthcare systems, although Swedish CGRP mAb access criteria are broadly consistent with those applied by other managed care organizations [39].

## Conclusions

In conclusion, our results support an overall sound medical rationale for CGRP mAb dispensations within Stockholm County. Treatment evaluation revealed that while national recommendations provided clear criteria for initiation and continuation, guidance on switching between CGRP mAbs was lacking. Our findings show that switching, particularly between erenumab and fremanezumab, was common, yet persistence declined across successive treatment sequences. This pattern, along with limited evidence supporting multiple switches, highlights the need for more structured guidance.

Importantly, our results can inform clinical decision-making by encouraging a more cautious approach to switching, helping clinicians limit unnecessary treatment transitions and optimize resource use.

## Electronic supplementary material

Below is the link to the electronic supplementary material.

Supplementary material 1

## Data Availability

The data presented in this study are not publicly available. Data can be requested from the Center for Health Data (Centrum för Hälsodata) at Region Stockholm, following ethical approval.

## Abbreviations

ATC: Anatomical Therapeutic Chemical classification system
BTX-A: Onabotulinumtoxin A (ATC code M03AX01)
CGRP: Calcitonin Gene-Related Peptide
CGRP mAbs: Monoclonal antibodies targeting the CGRP pathway
DDD: Defined Daily Dose
mAbs: Monoclonal antibodies
Region Stockholm: The regional public body responsible for healthcare within Stockholm County
SmPC: Summary of Product Characteristics
VAL database: Region Stockholm Healthcare Data Warehouse (VårdAnalysdataLager)
WHO ATC/DDD: The World Health Organization’s Anatomical Therapeutic Chemical (ATC) classification system and Defined Daily Dose (DDD)

## Declaration

### Conference presentations

An earlier version of this study was presented at the European Drug Utilization (EuroDURG) Conference, Uppsala, July 2025 (Abstract number 107, Abstract book page 193, URL: https://www.uu.se/en/department/pharmacy/research/pharmacoepidemiology/eurodurg-2025/abstract-book), and at the 19th European Headache Congress (EHC), Lisbon, December 2025 (Abstract number P399, Abstract book page 245, URL: https://headache-congress.org/programme-abstracts/abstract-booklet-2025/https://headache-congress.org/programme-abstracts/abstract-booklet-2025/).
