# Supplementary material for: Prescriber adherence to treatment guidelines for monoclonal antibodies against Calcitonin Gene-Related Peptide in migraine prophylaxis – a register-based cohort study
Source: J Headache Pain. 2026 Mar 10;27(1):78. doi: 10.1186/s10194-026-02322-1 (PMC12983619; doi:10.1186/s10194-026-02322-1)
Supplement: Supplementary file 1 — Supplementary material 1 [file 10194_2026_2322_MOESM1_ESM.docx]

# Supplementary material

## Supplementary - Results

**Supplementary table 1**. Number of days between CGRP mAbs dispensations (gaps) for individuals with more than one dispensation (n=2,154).

|  | **Mean** | **SD** | **Median** | **IQR** | **25 pct** | **75 pct** | **Min time** | **Max time** |
| --- | --- | --- | --- | --- | --- | --- | --- | --- |
| All CGRP mAbs | 38.9 | 33.0 | 30 | 15 | 26 | 41 | 1 | 910 |
| Erenumab | 38.6 | 34.3 | 30 | 14 | 26 | 40 | 1 | 910 |
| Fremanezumab | 39.6 | 30.6 | 31 | 15 | 27 | 42 | 1 | 559 |
| Galcanezumab | 38.5 | 27.9 | 31 | 13 | 27 | 40 | 1 | 374 |

**Supplementary table 2**. Number of CGRP mAb syringes per dispensation.

|  | **Mean** | **SD** | **Min** | **Max** |
| --- | --- | --- | --- | --- |
| All CGRP mAbs | 1.3 | 0.7 | 1 | 17 |
| Erenumab | 1.3 | 0.7 | 1 | 12 |
| Fremanezumab | 1.3 | 0.7 | 1 | 17 |
| Galcanezumab | 1.5 | 0.7 | 1 | 8 |


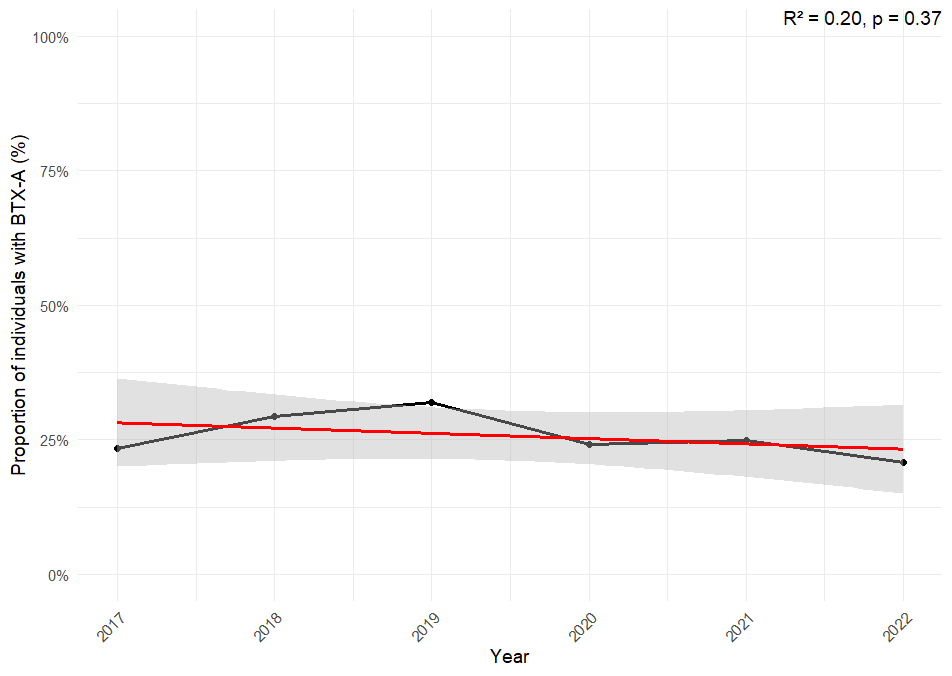


**Supplementary figure 1**: Annual proportion of individuals with CGRP mAb treatment (n = 2,266) who also received BTX‑A between 2017 and 2022. No statistically significant linear trend was observed with simple linear regression (p = 0.37).

### Sensitivity analyses: persistence with grace period 60 and 180 days


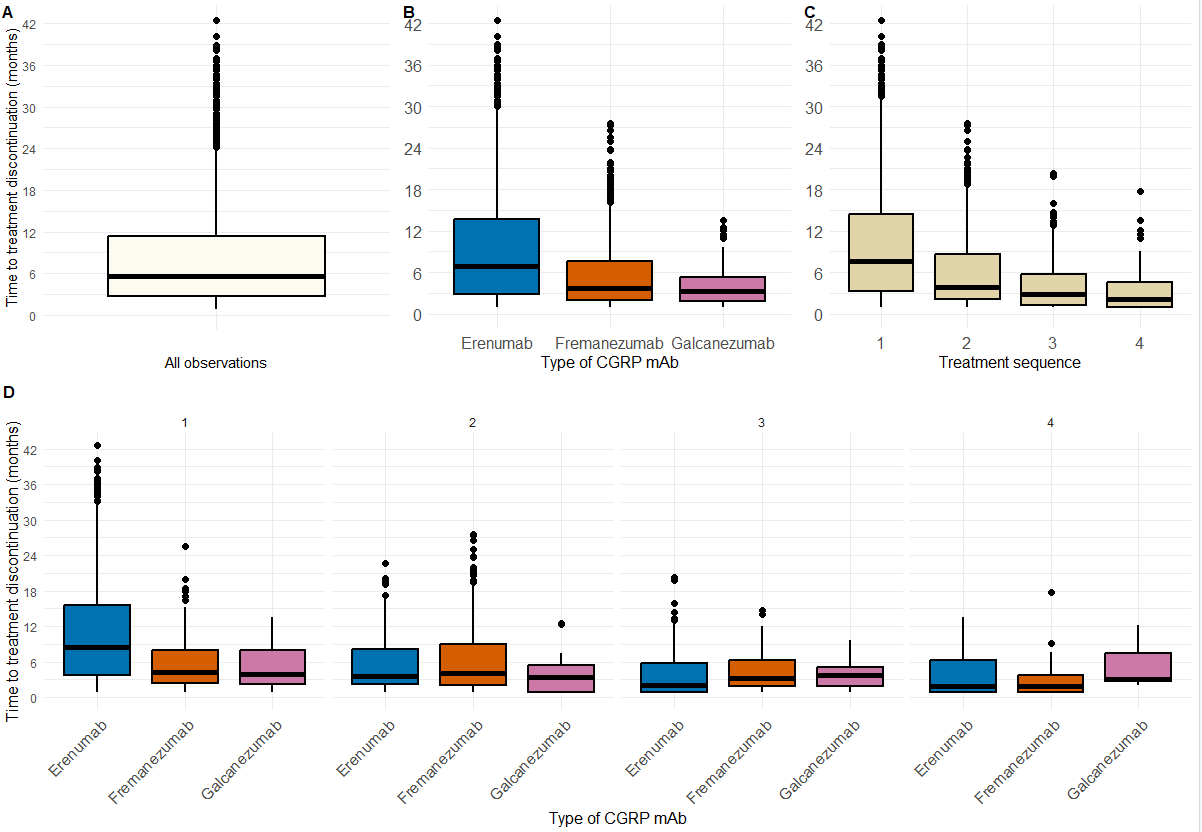


**Supplementary figure 2:** Boxplots (**grace period 60 days**) illustrating the distribution of time to treatment discontinuation of CGRP mAbs for those individuals who had experienced at least one treatment discontinuation (n = 1,419). **A**) for all CGRP mAbs and treatment sequences; **B**) for each type of CGRP mAb in all treatment sequences; **C**) for all CGRP mAbs in the first four treatment sequences; and **D**) for each type of CGRP mAb and for each treatment sequence (the first four). The thick horizontal line inside each box represents the median time to treatment discontinuation. The boxes extend from the first quartile (Q1) to the third quartile (Q3). The Interquartile Range (IQR = Q3–Q1), which covers the middle 50% of the data. The lines (whiskers) extending from the boxes indicate the range of data within 1.5 times the IQR from Q1 and Q3. The small points outside the whiskers represent outliers, which are values that fall beyond 1.5 times the IQR from the quartiles.


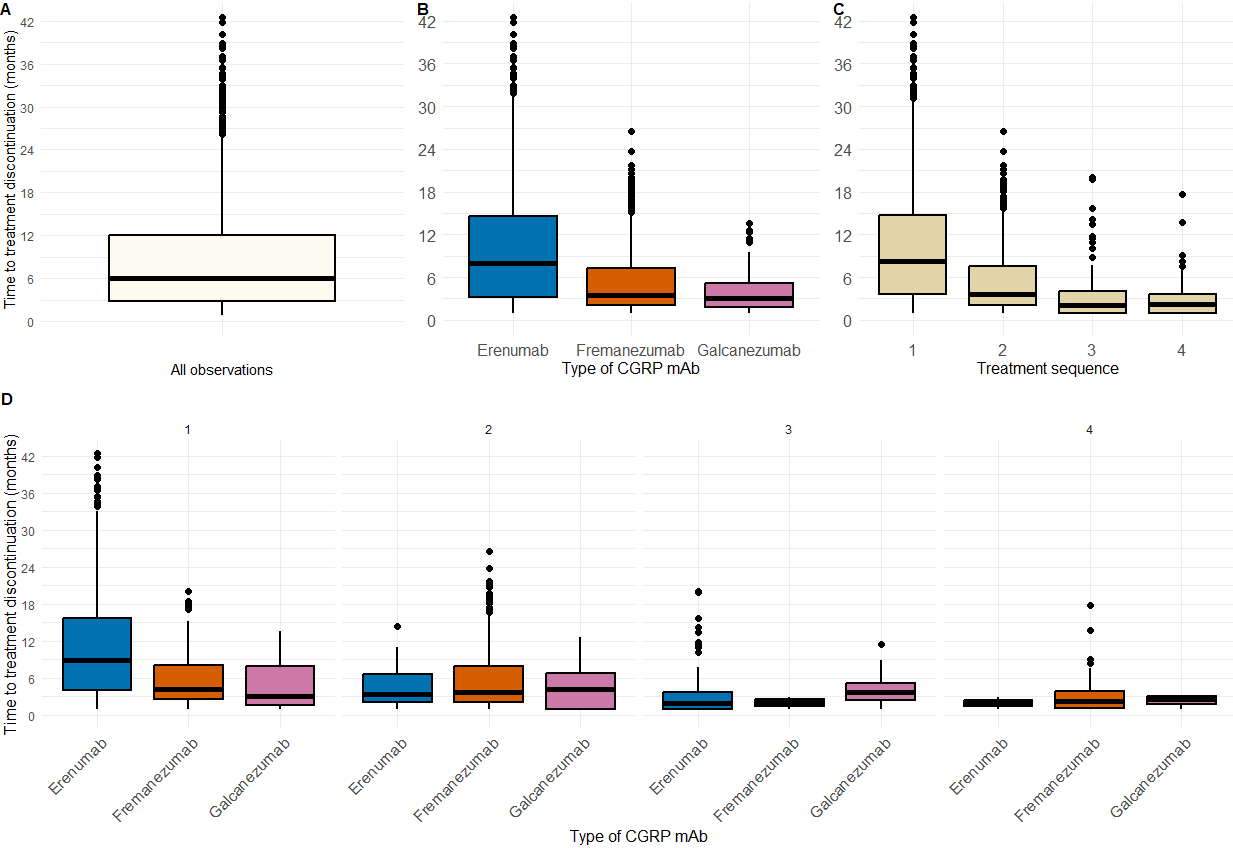


**Supplementary figure 3:** Boxplots (**grace period 180 days**) illustrating the distribution of time to treatment discontinuation of CGRP mAbs for those individuals who had experienced at least one treatment discontinuation (n = 1,182). **A**) for all CGRP mAbs and treatment sequences; **B**) for each type of CGRP mAb in all treatment sequences; **C**) for all CGRP mAbs in the first four treatment sequences; and **D**) for each type of CGRP mAb and for each treatment sequence (the first four). The thick horizontal line inside each box represents the median time to treatment discontinuation. The boxes extend from the first quartile (Q1) to the third quartile (Q3). The Interquartile Range (IQR = Q3–Q1), which covers the middle 50% of the data. The lines (whiskers) extending from the boxes indicate the range of data within 1.5 times the IQR from Q1 and Q3. The small points outside the whiskers represent outliers, which are values that fall beyond 1.5 times the IQR from the quartiles.


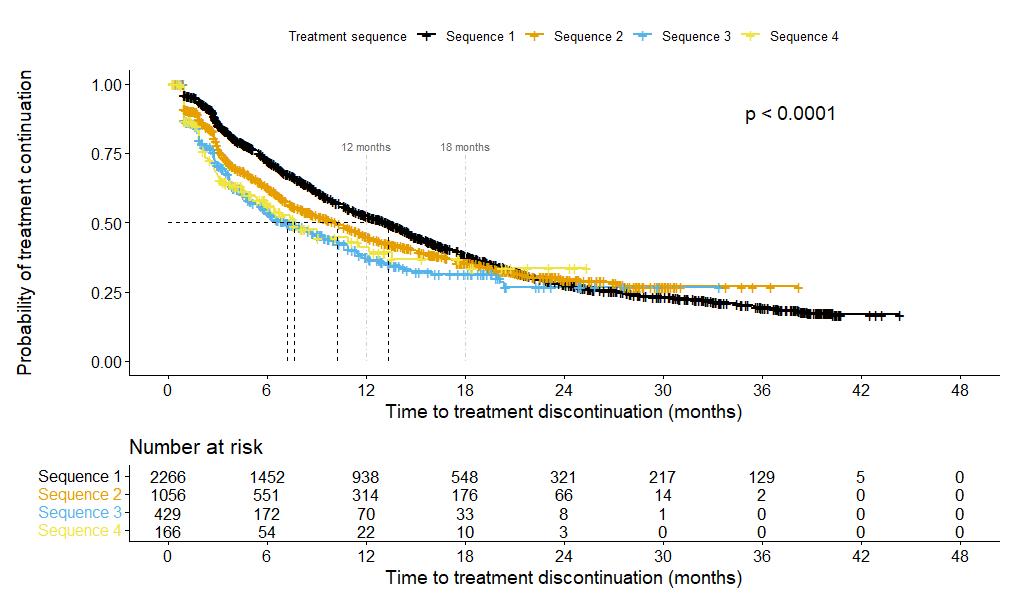


**Supplementary figure 4:** Kaplan Meier survival analyses (**grace period 60 days**) showing the probability of treatment continuation across up to four treatment sequences among all individuals who initiated a given sequence (n = 2,266). Log-rank p-value < 0.0001. Individuals without any treatment discontinuation before end of follow up were censured. The black line represents sequence 1, the orange line sequence 2, the blue line sequence 3, and the yellow line sequence 4. Black dashed lines represent the median survival time (50%) for each sequence. Light gray dashed lines at 12 and 18 months indicate the recommended time point for a treatment break following a positive response to CGRP mAbs.

**
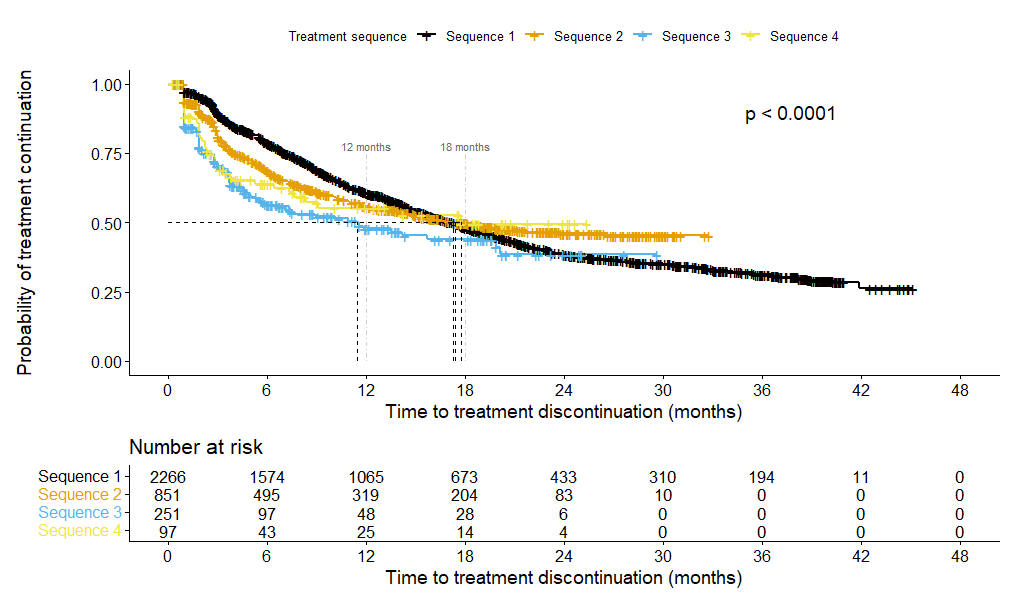
**

**Supplementary figure 5:** Kaplan Meier survival analyses (**grace period 180 days**) showing the probability of treatment continuation across up to four treatment sequences among all individuals who initiated a given sequence (n = 2,266). Log-rank p-value < 0.0001. Individuals without any treatment discontinuation before end of follow up were censured. The black line represents sequence 1, the orange line sequence 2, the blue line sequence 3, and the yellow line sequence 4. Black dashed lines represent the median survival time (50%) for each sequence. Light gray dashed lines at 12 and 18 months indicate the recommended time point for a treatment break following a positive response to CGRP mAbs.


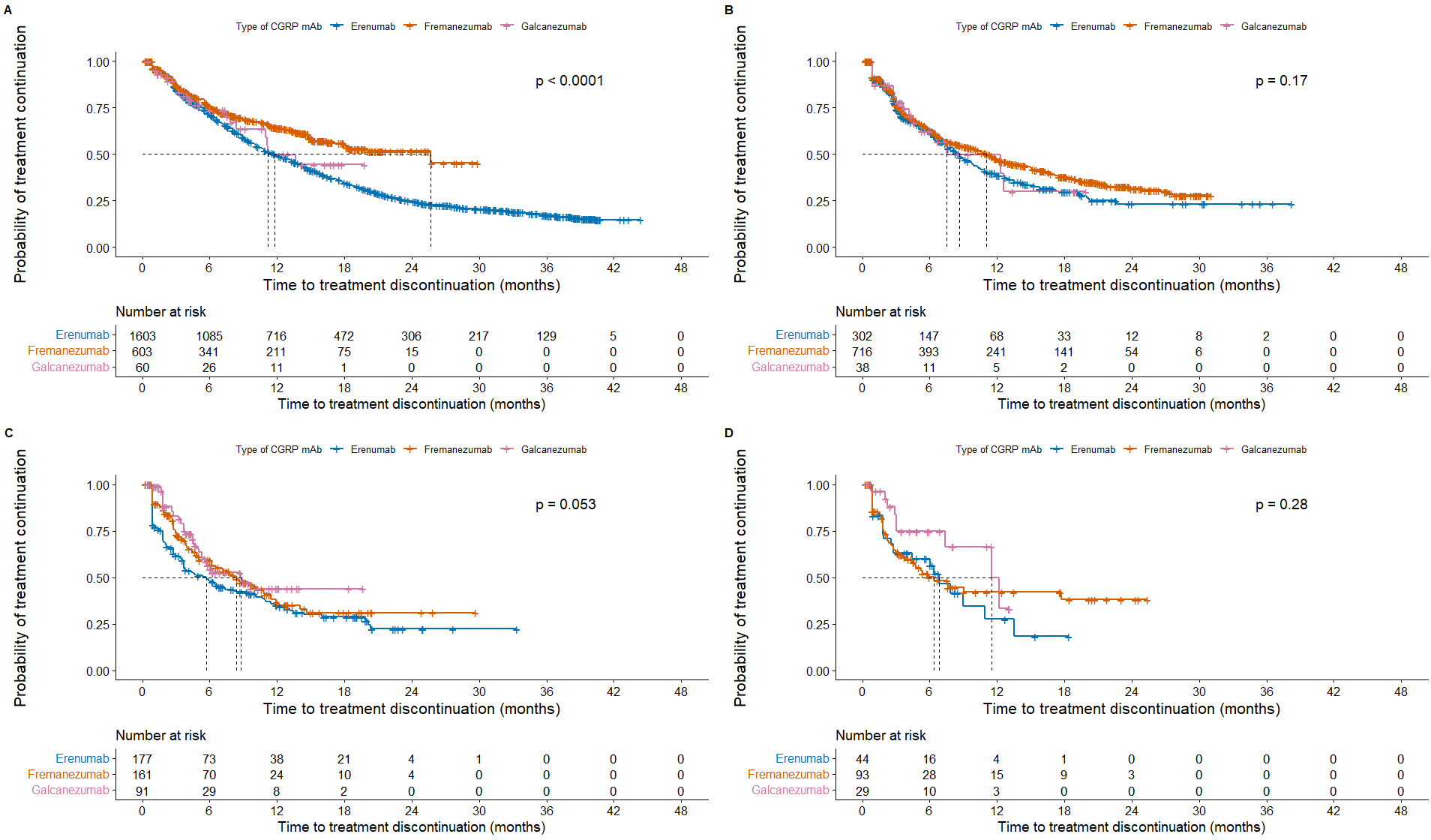


**Supplementary figure 6:** Kaplan Meier survival analyses (**grace period 60 days**) showing the probability of treatment continuation per type of CGRP mAb for **A)** treatment sequence 1 (n = 2,266), Log-rank p-value < 0.0001; **B)** treatment sequence 2 (n = 1,056), Log-rank p-value = 0.64; **C)** treatment sequence 3 (n = 429), Log-rank p-value < 0.0001; and **D)** treatment sequence 4 (n = 166), Log-rank p-value = 0.17. Individuals without any treatment discontinuation before end of follow up were censored. Blue lines represent erenumab, red lines represent fremanezumab, and pink lines represent galcanezumab. Black dashed lines represent the median survival time (50%) for CGRP mAb.


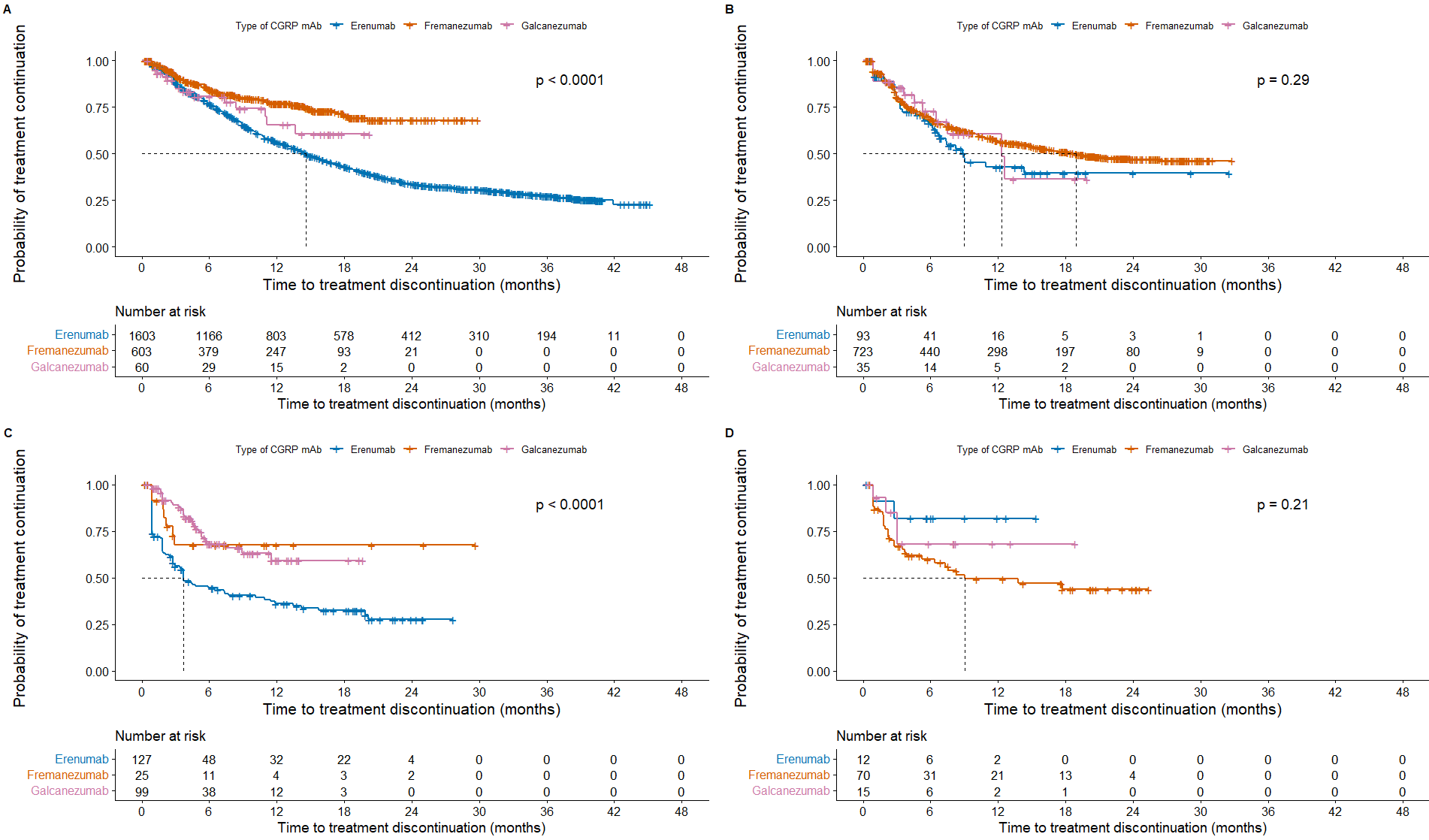


**Supplementary figure 7:** Kaplan Meier survival analyses (**grace period 180 days**) showing the probability of treatment continuation per type of CGRP mAb for **A)** treatment sequence 1 (n = 2,266), Log-rank p-value < 0.0001; **B)** treatment sequence 2 (n = 851), Log-rank p-value = 0.64; **C)** treatment sequence 3 (n = 251), Log-rank p-value < 0.0001; and **D)** treatment sequence 4 (n = 97), Log-rank p-value = 0.17. Individuals without any treatment discontinuation before end of follow up were censored. Blue lines represent erenumab, red lines represent fremanezumab, and pink lines represent galcanezumab. Black dashed lines represent the median survival time (50%) for CGRP mAb.


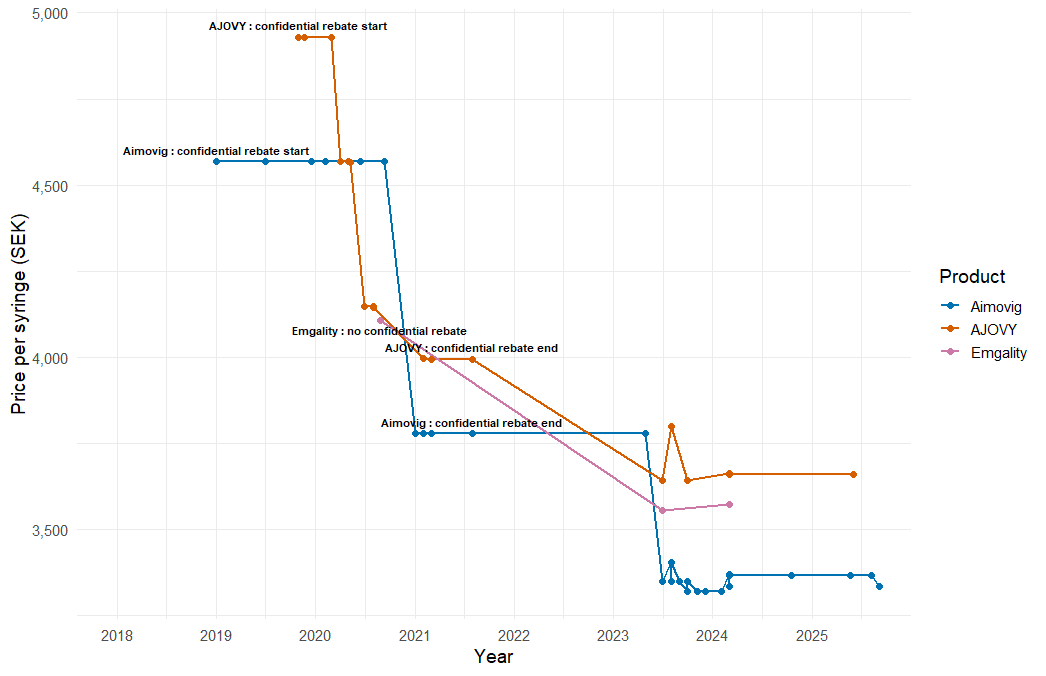


**Supplementary figure 8:** Official list price per syringe over time for each product. Data source: The Dental and Pharmaceutical Benefits Agency’s price and decision database. Note: the y-axis does not start at zero.


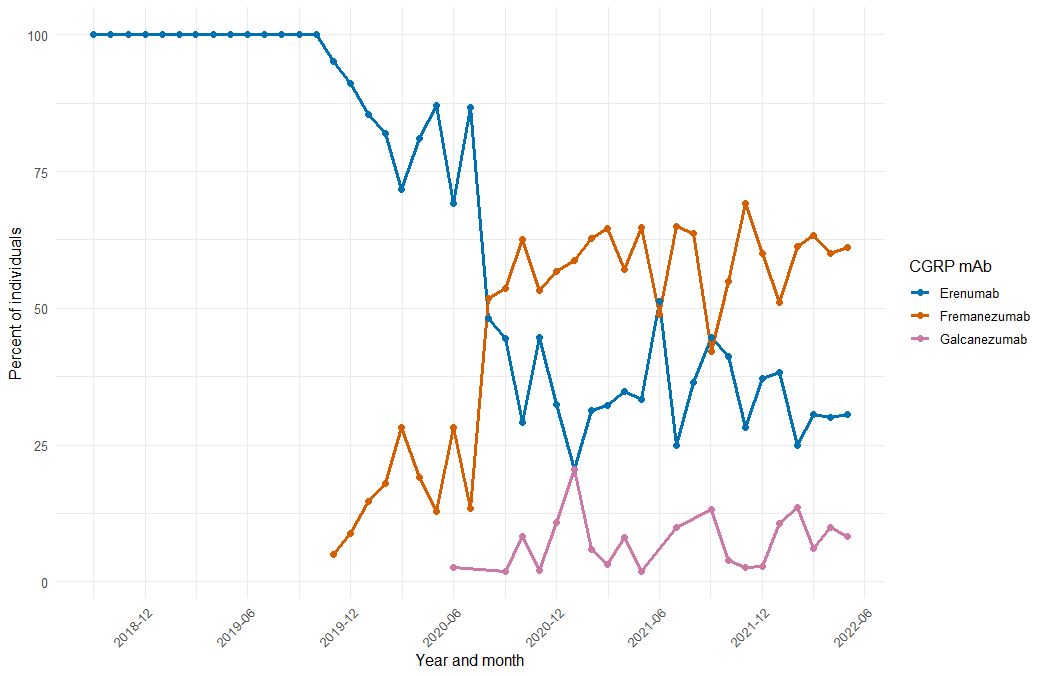


**Supplementary figure 9**: Monthly proportion of new users for each type of CGRP mAb (n=2,266).

## Supplementary – methodology

### Evaluation of treatment guidelines

We categorized the treatment guidelines into four themes: 1) patient eligibility; 2) prescriber qualifications; 3) treatment evaluation; and 4) monitoring and reporting. Each category was evaluated using one or several outcome variables.

#### Patient eligibility

Patient eligibility was evaluated by 1) the proportion of individuals with a migraine diagnosis (because of the lack of a specific ICD code for chronic migraine); 2) the proportion of individuals with both CGRP mAb dispensations and ATC code registrations for BTX-A from healthcare visits (BTX-A use indicates chronic migraine management, as Botox® has been approved for this condition since 2011); and 3) the proportion of individuals with a first CGRP mAb dispensation and prior dispensations of at least two other migraine prophylactic drugs at any time before or during the previous 12 months (corresponding to the recommendation that CGRP mAbs should be prescribed only to patients who have not responded adequately to at least two other prophylactic drugs). A sensitivity analysis of the annual proportion of individuals with CGRP mAb treatment who also received BTX A between 2017 and 2022 was conducted using simple linear regression.

#### Prescriber qualifications

Prescriber qualifications were assessed by the percentage of individuals in the study population whose CGRP mAb prescription originated from prescribers with workplace codes categorized as ‘Neurology or Headache’ in accordance with recommendations that CGRP mAbs should be prescribed exclusively by neurologists or physicians practicing in specialized migraine clinics.

#### Treatment evaluation

To estimate the practice of CGRP mAb treatment evaluation, we conducted several analyses on treatment persistence using the refill-gap method (1) and time to treatment discontinuation. Overlapping dispensations (i.e., early refills) were ignored, assuming patients started using the subsequent dispensation immediately upon its dispensation date, without considering the remaining supply from the previous dispensation or extending the treatment duration. To apply this assumption consistently, we calculated the number of days covered by each CGRP mAb dispensation based on dosage information from the Summary of Product Characteristics (SmPC) and the DDDs according to the WHO ATC/DDD system. Each pre-filled syringe or pen of erenumab (70 mg or 140 mg), fremanezumab (225 mg), and galcanezumab (120 mg) was assumed to cover a 28-day period. Here, we did not adjust for the galcanezumab loading dose (240 mg), as it occurs at the start of treatment.

##### Definition of Treatment Discontinuation and Grace Periods

Treatment discontinuation for each dispensed type of CGRP mAb (erenumab, fremanezumab, or galcanezumab) was defined using the following criteria: 1) a gap exceeding the number of days supplied by the previous dispensation plus an additional 122-day (about 4 months) grace period between two dispensations; 2) a final dispensation with no subsequent refill, where the gap between the last dispensation and the study end date (June 9, 2022) exceeded the number of days supplied by the final dispensation, plus an additional 122-day grace period; or 3) a switch from one type of CGRP mAb to another. A treatment sequence was defined as a continuous period during which an individual is on one type of CGRP mAb without a treatment discontinuation or a switch.

The 122-day grace period was chosen based on clinical and pharmacological considerations and to align with a previous CGRP mAb persistence analysis by the Dental and Pharmaceutical Benefits Agency (2). It reflects the dose frequency (monthly or quarterly) and the long elimination half-life of CGRP mAbs, approximately 30 days. After discontinuation, the drug remains in the body for about 150 days (5 half-lives) and its effects may persist for months. The grace period accounts for this prolonged effect, especially for quarterly dosing regimens, such as fremanezumab. Two alternative grace periods, 60 and 180 days, were used in a sensitivity analysis to test the robustness of the results.

##### Visualization of Treatment Persistence

Boxplots were generated to visualize the distribution of time to treatment discontinuation among individuals who had experienced at least one discontinuation. Four sets of boxplots were created: (1) all CGRP mAbs and treatment sequences combined, (2) stratified by CGRP mAb type, (3) stratified by the first four treatment sequences, and 4) stratified by both CGRP mAb type and treatment sequence. Statistical differences between group medians were assessed using the non-parametric Kruskal–Wallis test, as assumptions of normality and homogeneity of variance were violated, followed by Dunn’s post hoc test for pairwise comparisons with Bonferroni correction.

Moreover, a Sankey diagram was created to illustrate transitions between treatment sequences, including switches between CGRP mAb types and discontinuations, which could represent either complete treatment cessation or discontinuation followed by reinitiation of the same CGRP mAb type. The analysis included only individuals who had experienced at least one treatment discontinuation, and a total of five sequences were considered.

Finally, Kaplan-Meier survival analyses were performed to evaluate treatment persistence over time. We evaluated the probability of continued treatment during the first four treatment sequences, both overall and stratified by CGRP mAb type. Censoring occurred for individuals who had not discontinued treatment before the end of follow up. Kaplan-Meier curves were generated to visualize the probability of continued treatment, with accompanying risk tables showing the number of individuals at risk at each time point. Log-rank tests were used to assess statistical differences in persistence across treatment sequences and between CGRP mAb types.

#### Monitoring and Reporting

The monitoring and reporting aspects of the CGRP mAb treatment recommendations were evaluated by comparing the number of individuals recorded in the register *Severe Neurovascular Headache* as newly initiated on CGRP mAbs during the study period with the number of CGRP mAb-treated individuals in our study population. This comparison reflects the monitoring and quality control aspect of migraine treatment within the healthcare system.

**References**

1. Rasmussen L, Wettermark B, Steinke D, Pottegard A. Core concepts in pharmacoepidemiology: Measures of drug utilization based on individual-level drug dispensing data. Pharmacoepidemiol Drug Saf. 2022;31(10):1015-26.

2. The Dental and Pharmaceutical Benefits Agency. Pharmaceutical Benefits Board. Documentation for Reimbursement Decision – Re-evaluation of CGRP Inhibitors. Case No. 1615/2022. [Swedish] 2023 [Available from: <https://www.tlv.se/lakemedelsforetag/omprovning-av-lakemedel/arkiv-avslutade-omprovningar-lakemedel/2023-06-19-cgrp-hammarna-kvarstar-i-hogkostnadsskyddet-till-sankt-pris-med-oforandrad-subventionsbegransning.html>.
